# Assessment of Solitary Pulmonary Nodules Based on Virtual Monochrome Images and Iodine-Dependent Images Using a Single-Source Dual-Energy CT with Fast kVp Switching

**DOI:** 10.3390/jcm9082514

**Published:** 2020-08-04

**Authors:** Arkadiusz Zegadło, Magdalena Żabicka, Marta Kania-Pudło, Artur Maliborski, Aleksandra Różyk, Witold Sośnicki

**Affiliations:** 1 Department of Radiology, Military Institute of Medicine, Szaserów 128, 04-141 Warsaw, Poland; mzabicka@wim.mil.pl (M.Z.); mkania-pudlo@wim.mil.pl (M.K.-P.); amaliborski@wim.mil.pl (A.M.); 2 Department of General, Oncological, Metabolic and Thoracic Surgery, Military Institute of Medicine, Szaserów 128, 04-141 Warsaw, Poland; wsosnicki@wim.mil.pl

**Keywords:** gemstone spectral imaging, dual-energy CT, virtual monochromatic images, iodine map, spectral Hounsfield unit attenuation curves, solitary lung tumor, lung cancer

## Abstract

With lung cancer being the most common malignancy diagnosed worldwide, lung nodule assessment has proved to be one of big challenges of modern medicine. The aim of this study was to examine the usefulness of Dual Energy Computed Tomography (DECT) in solitary pulmonary nodule (SPN) assessment. Between January 2017 and June 2018; 65 patients (42 males and 23 females) underwent DECT scans in the late arterial phase (AP) and venous phase (VP). We concluded that imaging at an energy level of 65 keV was the most accurate in detecting malignancy in solitary pulmonary nodules (SPNs) measuring ≤30 mm in diameter on virtual monochromatic maps. Both virtual monochromatic images and iodine concentration maps prove to be highly useful in differentiating benign and malignant pulmonary nodules. As for iodine concentration maps, the analysis of venous phase images resulted in the highest clinical usefulness. To summarize, DECT may be a useful tool in the differentiation of benign and malignant SPNs. A single-phase DECT examination with scans acquired 90 s after contrast media injection is recommended.

## 1. Introduction

Solitary pulmonary nodule (SPN) assessment is one of the major challenges of modern medicine.

With over 14 million malignancies diagnosed worldwide in 2012, lung cancer accounted for approximately 13% (1.8 million) of these malignancies. Lung cancer is the most common cancer diagnosed in men (16.7% of the total) and the third most common in women (8.7% of the total), after breast and colorectal cancer. It is also the leading cause of cancer death in both sexes combined (1.6 million deaths, representing 20% of total cancer mortality) and in the male population (23.6% of the total). For the female population, it accounts for 13.8% of cancer deaths, being surpassed only by breast cancer (14.7% of total) [1]

The pulmonary nodule is defined as a round or irregular opacity, which may be well or poorly defined and no larger than 3 cm on its maximum diameter. The management of incidental pulmonary nodules was described in the Fleischner Society guidelines published in 2017 [2]. Contrast-enhanced conventional computed tomography (CCT) of the thorax is often the initial radiological modality used to confirm the clinical suspicion of a lung tumor [3]. Positron emission tomography with fluorine 18-labelled fluorodeoxyglucose (^18^ F-FDG PET) facilitates the differentiation of SPNs, especially solid ones and those with a diameter of 10 mm or more [4]. Older age, a history of smoking, and the growth of a nodule increases the risk of malignancy [2,4]. The imaging biomarkers used in the assessment of a lung nodule include location, size, boundaries, heterogeneity [4], and the degree of tissue contrast enhancement [5].

The degree of contrast enhancement of the SPN is the difference between the amount of the absorbed X-radiation by the tumor before and after intravenous contrast administration, with an increase of ≥15–20 Hounsfield units (HU) indicating malignancy [6]. Patients examined using CCT are scanned with 120 kVp X-rays possessing a polychromatic spectrum [7]. A beam-hardening artifact, which occurs when an X-ray beam passes through objects of varying densities, such as metal and soft tissues, may be easily misinterpreted as disease [8].

**Figure 1:**
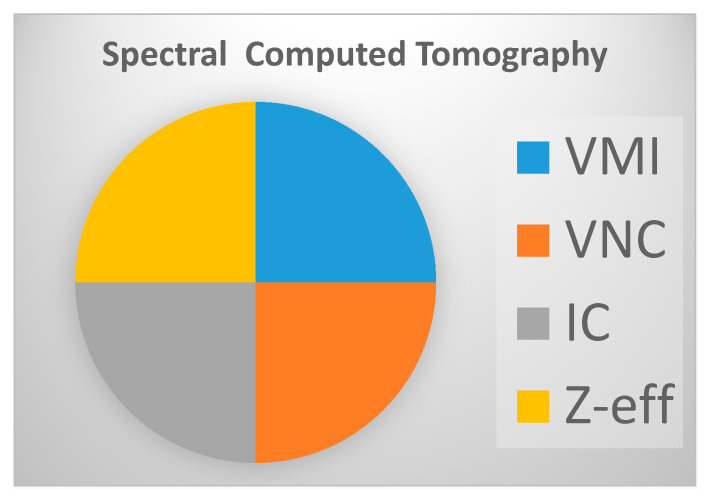
Imaging possibilities in Dual Energy Computed Tomography (DECT). VMI—virtual monochromatic images; VNC—virtual non contrast; IC—iodine concentration; Z-eff—analysis based on effective atomic number.

Dual Energy Computed Tomography (DECT) was first reported in 1977 [9] but was not used clinically until 2006 due to technical challenges, and promises to be the solution to this problem. Imaging possibilities of DECT are presented in Figure 1. The use of X-ray beams at two different kVp energy levels, low (80 kVp) and high (140 kVp), reduces or eliminates beam-hardening artifacts [10]. The use of virtual monoenergetic images (VMI) also decreases pseudoenhancement artifacts, providing more accurate density measurements [11,12]. Spectral DECT using quantitative analyses of iodine concentration (IC) maps and virtual monoenergetic images (VMI) facilitates the assessment of possible malignant lesions, including solitary pulmonary nodules (SPNs) [13,14].

The key advantage of DECT is that it obtains material attenuation differences at two X-ray energy levels. These spectral data are further postprocessed and used to perform tissue characterization [15]. Sudarski et al. in their review concerning the clinical utility of DECT in the evaluation of pulmonary masses and nodules presented this method as very promising, offering not only morphological but also functional information [16].

The aim of our study is to demonstrate the clinical usefulness of VMI and IC images in differentiating solitary pulmonary nodules of no more than 30 mm in diameter.

## 2. Materials and Methods

### 2.1. Patients

The Bioethics Committee of the Military Institute of Medicine (4/WIM/2017) in Warsaw, Poland, approved this prospective study. All the patients provided written informed consent. Between January 2017 and June 2018, DECT scans were performed in 65 patients—42 males and 23 females (median age was 67 years; range 31–83 years). The inclusion and exclusion criteria are presented in Table 1.

The interpreting radiologist at the time of the primary reading had access to the patients’ clinical data but had no knowledge of the histopathological results of the examined SPNs because those results were obtained after the DECT examinations. In order to confirm the diagnoses, patients with benign lesions underwent an additional 12-month clinical and imaging follow-up observation.

### 2.2. DECT Examination

The patients underwent examinations on a single source DECT scanner with rapid kVp switching Discovery CT 750 HD (GE Healthcare, WI, USA) in the late arterial phase (AP) and venous phase (VP) with delay times after contrast media injections of 35 and 90 s, respectively. The following other DECT parameters were set: tube voltage range, 80 kVp–140 kVp; tube rotation time, 0.5 s; tube current, 630 mA; helical pitch, 1.375:1; small field of view (SFOV), 50.0 cm; collimation, 55 mm; and slice thickness, 0.625 mm. A nonionic contrast medium (400 mg/mL, 1 mL/kg, Iomeron, Bracco Imaging Deutschland GmbH based in Konstanz, Germany) was administered to each patient at a rate of 4.0 mL/s using a power injector.

### 2.3. Data Analysis

The CT scans were analyzed using the AW VolumeShare 7 (GE Healthcare) diagnostic station. ROIs (regions of interest) were placed on the relatively homogeneous areas of the nodules, which were assessed in the late-arterial (AP) and venous (VP) phases of contrast enhancement on monochromatic images and iodine concentration images using the copy-and-paste function. The nodule size was measured in the long axis of the lesion on the lung window setting, as recommended [16,17]. The mean values of the absorbed X-radiation in SPNs were measured on VMS (virtual monochromatic spectral) maps in both phases (AP and VP) in the energy range from 40 to 140 keV.

Images and 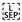 quantitative parameters were measured at the workstation (AW 4.7; GE Healthcare) in the AW VolumeShare 7 protocol on general maps (window parameters W: 1500, L: −600).

**Figure 2:**
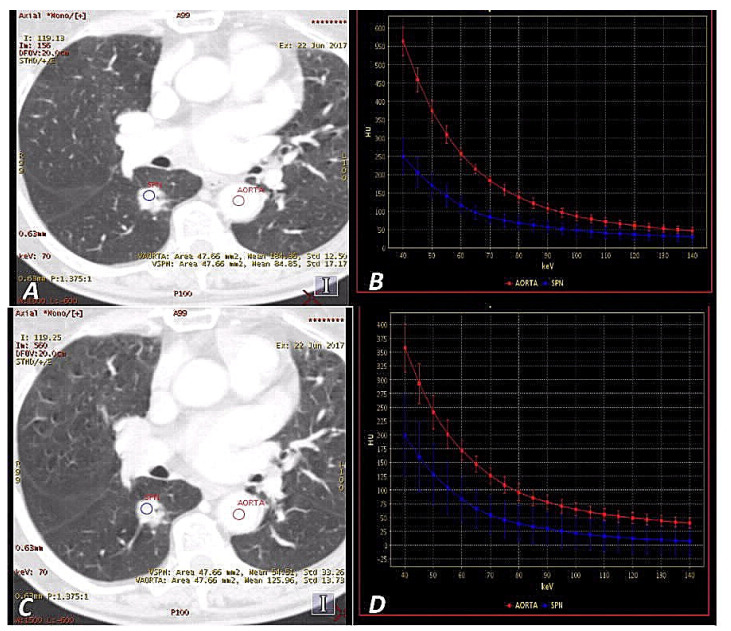
A 65-year-old woman with adenocarcinoma in AP (late-arterial phase) (**A**,**B**) and VP (venous phase) (**C**,**D**). Monochromatic 70 keV _SS_DECT (single source Dual Energy Computed Tomography) images show a hyperattenuating solitary pulmonary nodule(SPN) in the right lung (blue regions of interest (ROI)) (**A**,**C**). The graphs show DECT spectral attenuation curves for the enhancing mass SPN (blue) and descending aorta (red) (**B**,**D**). An increasing curve slope is visible at lower energies, which is related to the interaction of iodine atoms with photons at these energy levels based on the photoelectric effect. Attenuation of SPN mean: (AP) = 84.85 HU, (VP) = 54.51 HU.

**Figure 3:**
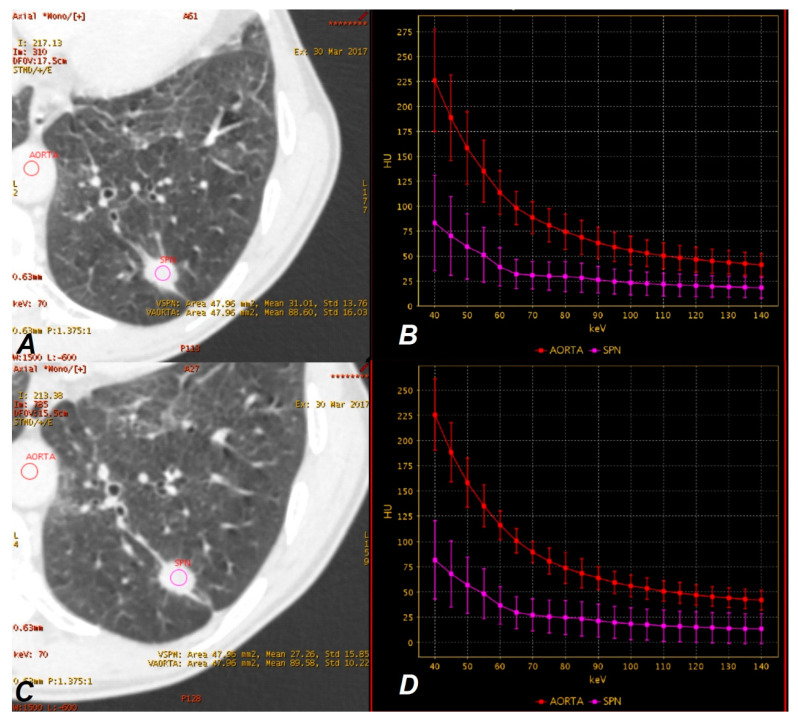
A 60-year-old man with tuberculoma in AP (**A**,**B**) and VP (**C**,**D**). Monochromatic 70 keV _SS_DECT images show an attenuating SPN on the left lung (purple ROI) (**A**,**C**). Graphs show DECT spectral attenuation curves for the enhancing mass SPN (purple) and descending aorta (red) (**B**,**D**). The smaller slope of the spectral curve and lowering of the position than in Figure 2b,d is due to the lower accumulation of iodine in the tumor. Attenuation SPN mean: (AP) = 31.01 HU, (VP) = 27.26 HU.

The measurements in the ROI area (with the “auto-plot” function enabled) included the mean value of absorbed radiation (HU), standard deviation (HU) and area of measurement (mm^2^). Examples are displayed in Figure 2 and Figure 3.

**Figure 4:**
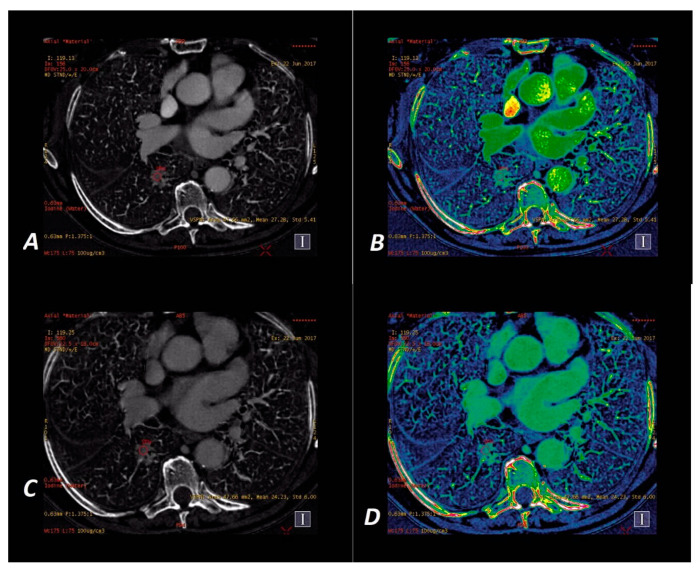
A 65-year-old woman with adenocarcinoma. Iodine–based _SS_DECT images in AP (**A**,**B**) and VP (**C**,**D**). Iodine material density ssDECT image shows the iodine content in the region that shows hyperattenuation in SPN on Figure 2a,c. Colormap “French” (**B**,**D**) helps to identify the tumor compared to their standard “Linear Gray” map (**A,C**). Iodine concentration mean = 27.28 × 100 µg/cm^3^ (AP) and 24.23 × 100 µg/cm^3^ (VP).

**Figure 5:**
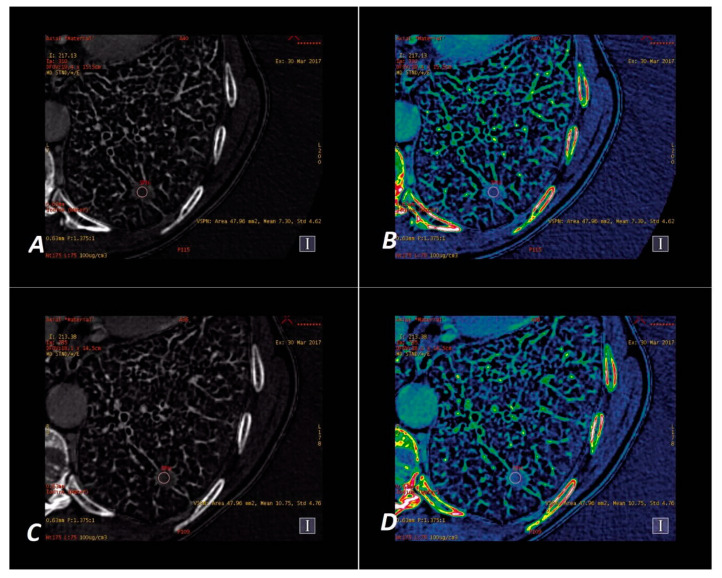
A 60-year-old man with tuberculoma. Iodine–based _SS_DECT images in AP (**A**,**B**) and VP (**C**,**D**). Iodine material density ssDECT image shows the iodine content in the region that shows hyperattenuation in SPN on Figure 3a,c. Colormap “French” (**B**,**D**) helps to identify the tumor compared to their standard presentation in “Linear Gray” (**A**,**C**). Iodine concentration mean = 7.30 × 100 µg/cm^3^ (AP) and 10.75 × 100 µg/cm^3^ (VP).

The iodine concentration in the SPNs was retrieved directly from the IC maps at the energy level of 70 kVp in the AP and VP phase of contrast enhancement on Mono/MD Review maps with the Colormap “French” overlay to increase the assessed lesion contrast. The spectral curves for X-ray absorption in the spectral scan were determined using the MD Analysis/Spectral HU Curve software via the insertion of two ROIs—one in the assessed lesion and the other in the descending aorta—thus determining the reference point for each measurement. Examples of malignant and benign nodules displayed on iodine concentration-based images are shown in Figure 4 and Figure 5.

### 2.4. Statistical Analysis

The STATISTICA 12.0 software was used for the statistical analyses of tumor size measurements, the contrast enhancement of SPNs on VMS maps in AP and VP, and the iodine concentration in SPNs on IC maps in AP and VP. All the SPNs evaluated on monochromatic images in the AP and VP of DECT scans were statistically analyzed in the energy range 80–140 keV at 5 keV intervals with the determination of threshold values that differentiate malignant and benign lesions. For the following variables with a normal distribution—“IC (AP)”, “IC (VP)”, “enhancement of SPNs (AP) i (VP)”, “VMS 75–140 keV (AP)”, and “60–110 keV (VP)”—the results are presented as the means and standard deviations for each of these variables, and the Student’s *t*-test was used to assess the statistical significance of differences between the malignant and benign nodules. Due to the non-normal distribution of “age”, “size”, and “SPN gain” on VMS (Virtual Monochromatic Spectral) 40–70 keV (AP) maps and VMS 40–55 keV (VP) and 115–140 keV (VP) maps, the results were reported as the median and range (minimum and maximum), and the Mann–Whitney U test was used to evaluate these variables.

The value of *p* < 0.05 was considered statistically significant.

Receiver operating characteristic (ROC) curves were used to determine the threshold values for differentiating the malignant and benign nodules and establishing the sensitivity and specificity values. Youden’s index was used to determine these values. The diagnostic performance was evaluated by calculating the area under the ROC curve (AUC). ROC and AUC analyses were performed for the measurements obtained on VMS (AP) maps and VMS (VP) maps. The best results (one for each phase of the study—AP and VP) were compared using ROC and AUC analyses of the iodine concentration measurements in SPNs in the AP and VP phases, respectively.

## 3. Results

### 3.1. Radiation Dose

We used a fixed scan protocol. Therefore, for each DECT scan the volumetric CT dose index (CTDIvol) was a fixed value of 12.72 mGy for each phase (AP and VP).

According to ACR (American College of Radiology) data, the radiation dose values are acceptable [18] but higher than the standard chest CT scan protocol that is routinely used in our institution (average conventional CT scan values are CTDIvol = 11.25 mGy and include one phase protocol after contrast enhancement).

### 3.2. Pathological Results

The histopathological assessment of SPNs was performed using the following diagnostic procedures: CT-guided biopsy (24 cases, 37%), surgery (21 cases, 33%), thoracoscopic lung biopsy (14 cases, 21%), percutaneous lung biopsy (4 cases, 6%), and Endobronchial Ultrasound-guided Transbronchial Needle Aspiration (EBUS-TBNA) (2 cases, 3%). Out of the 65 nodules examined using the DECT technique, 46 (71%) were malignant, including 23 cases of adenocarcinoma (35%) and 18 cases of squamous cell carcinoma (28%). Out of the 19 diagnosed benign nodules, inflammatory lesions were the largest group, with nine cases (14%). The results of the histopathological evaluation of nodules are presented in detail in Table 2.

### 3.3. DECT Imaging Results

The median size of benign SPNs was 19.00 mm (range 8.5–30 mm), which was not statistically significantly different from the median size of the malignant nodules, which were larger (the median size was 24.05 mm; range 9.80–30.00 mm; *p* = 0.07).

### 3.4. Results of the VMS Analysis

**Figure A1:**
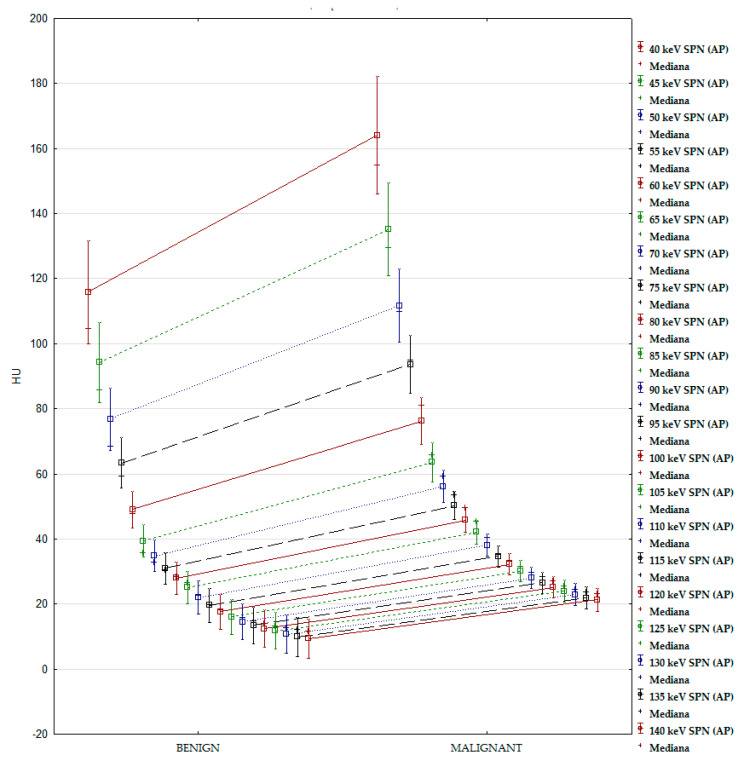
Distribution of the mean values of absorbed radiation measured on monochromatic images (AP) in benign and malignant nodules.

**Figure A2:**
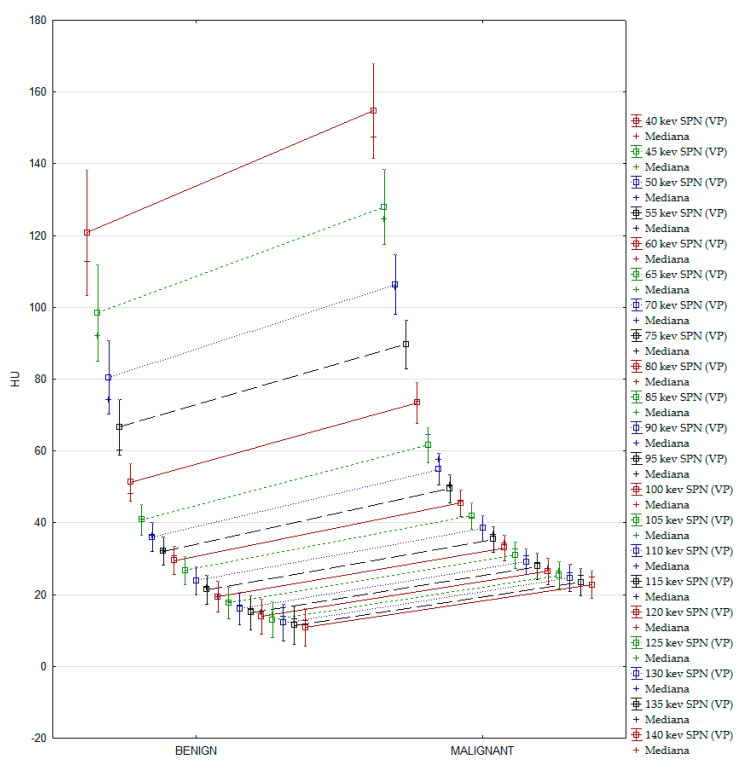
Distribution of the mean values of absorbed radiation measured on monochromatic images (VP) in benign and malignant nodules.

Pulmonary nodules showed a different distribution of the degree of X-ray beam absorption in the 40–140 keV energy spectrum on monochromatic images. Measurements were taken at 5 keV intervals. A graphical presentation of the results is shown in Figure A1 and Figure A2 (found in Appendix A). 

Statistical analysis of the VMS images showed statistically significant differences between the doses of radiation absorbed by the benign and malignant nodules in both phases of contrast enhancement, with the degree of radiation absorption in the malignant lesions being significantly higher in the entire X-ray spectrum in AP (Table A1 in Appendix A) and VP (Table A2 in Appendix A). Based on the AUC values, we selected monochromatic images with a photon energy of 65 keV with the largest areas under the ROC curves for further analyses.

### 3.5. Results of the IC Map Analyses

The IC maps showed significant differences in iodine concentrations in the benign and malignant lesions (*p* < 0.001) in both phases of contrast enhancement. In the malignant lesions, a significantly higher average iodine content was observed compared to the benign lesions in AP (19.72 × 100 vs. 12.63 × 100 µg/cm^3^) and VP (18.11 × 100 vs. 12.85 × 100 µg/cm^3^). IC (AP) and IC (VP) analyses are based on the ROC curves, with the determination of threshold values determining the sensitivity and specificity of the test and the AUC surface. The results are shown in Table 3.

The adopted values for differentiating malignant lesions from benign lesions were 14.84 × 100 µg/cm^3^ (AP) and 12.11 × 100 µg/cm^3^ (VP), with a diagnostic sensitivity and specificity of 87% and 74%, respectively (AP), and 96% and 63%, respectively (VP).

### 3.6. ROC Curves and AUC

The AUC for SPNs measured on 65 keV VMI and IC maps in the AP and VP showed very similar values, and the differences between the AUC 65 keV and AUC IC measurements in both contrast enhancement phases were not significant (*p* = 0.90 and r = 0.11 for AP; *p* = 0.56 and r = 0.13 for VP).

**Figure 6:**
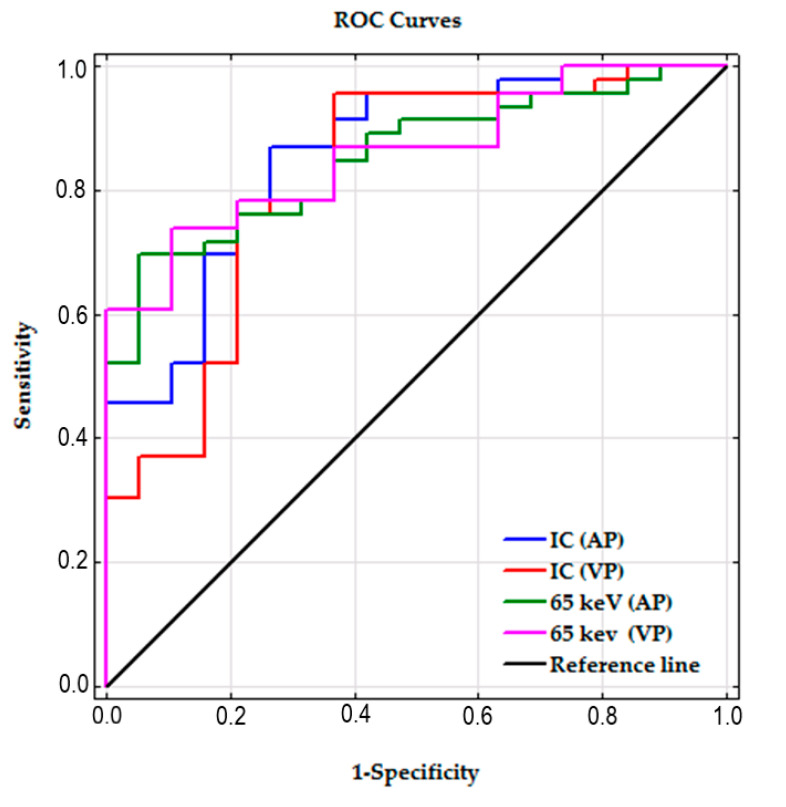
ROC curves of all the parameters used to differentiate the malignant and benign SPNs.

The ROC curves for all the analyzed parameters are shown collectively in Figure 6.

### 3.7. Clinical Implications

All four parameters—65-keV VMI (AP), 65-keV VMI (VP), IC (AP), and IC (VP)—proved useful in diagnosing the malignancy of pulmonary nodules examined in DECT. The differential analysis based on the absorbed radiation levels at 65 keV, which best represented the differences based on the AUC values on the ROC curves, allowed the diagnosis of malignancy in 32 out of 46 patients (70%) in AP and in 34 out of 46 patients (74%) in VP.

The established threshold values of 53.99 HU (AP) and 49.65 HU (VP) falsely diagnosed a malignancy in 1 out of 19 patients (5%) based on the assessment of the lesion in AP, and in 2 out of 21 patients (10%) after the assessment in VP. Among the 46 malignant nodules, 14 (30%) in AP had a density lower than 53.99 HU at an energy level of 65 keV, which did not allow their detection (6 cases of adenocarcinoma, 6 cases of squamous cell carcinoma, and 2 cases of large cell neuroendocrine carcinoma).

The examination failed to detect 12 out of 46 (26%) malignant tumors that had densities lower than 49.65 HU in the 65 keV VMI images in VP (6 cases of squamous cell carcinoma, 4 cases of adenocarcinoma, 2 cases of large cell neuroendocrine carcinoma).

After the analysis of the nodules on IC maps based on the calculated threshold values of 14.84 × 100 (AP) and 12.11 × 100 µg/cm^3^ (VP) (established using the Youden Index of ROC), the significant clinical usefulness of this method was demonstrated.

This method allowed the diagnosis of cancer in 40 out of 46 people (87%) after the examination in AP of DECT, and in 44 out of 46 patients (96%) in VP.

Malignancy was incorrectly diagnosed in 5 out of 19 people (26%) in the AP and in 7 out of 19 people (36%) in the VP (5 cases of inflammatory infiltrations, 1 case of sarcoidosis, and 1 case of hamartoma).

ROC analyses of the 65 keV VMI and IC parameters in both contrast enhancement phases showed their clinical usefulness and showed differences in the examined material (Table 4).

## 4. Discussion

Dual-energy CT is an advanced tool that uses two different energy settings simultaneously to study differences in tissue energy suppression at different energy levels. The photoelectric effect used to absorb photon energy is strongly dependent on the atomic number (Z) of the tissue. Most commonly occurring atoms, such as oxygen (Z = 8), nitrogen (Z = 7), carbon (Z = 6), and hydrogen (Z = 1), have a small number of protons in the nucleus, which results in a weak photoelectric effect. Therefore, to increase the probability of photoelectric events in tissues, contrast agents constructed of atoms with a high atomic number, such as iodine (Z = 53), are used.

The photoelectric effect is directly dependent on the energy that must be supplied to produce it (the so-called K-edge value), which is 33.2 keV for the iodine atom [19]. In routine CT scans using a polychromatic X-ray beam, tissue attenuation is based largely on the Compton effect, which, unlike the photoelectric effect, does not depend on the atomic number of the absorber.

The diagnosis of lung tumors in CCT is based primarily on the assessment of morphology and the degree of contrast enhancement, which is the difference between the density of a tissue after contrast administration and its native density. A tumor enhancement ≥ +15–20 HU indicates a high probability of malignancy, with a sensitivity of 95–100% and specificity of 58–93% [6]. Twenty Hounsfield units was also found to be the optimal threshold for defining enhancement in DECT examinations of pulmonary nodules of less than 3 cm on size with a 100% sensitivity and 71% specificity [20].

Virtual monochromatic images are based on algorithms developed by Alvarez and Macovski [21]. Scanning in ssDECT is conducted at two energy levels, 80 and 140 keV, using a single detector (Gemstone Spectral Imaging). A tumor is assessed by evaluating the amount of absorbed radiation at individual energy levels, which allows the creation of spectral curves. The images “correlate” with the amount of radiation absorbed by the tissue at any given energy level of a photon. This process allows for subjective assessments of images and the selection of optimal parameters for the presented pathology, which is not possible in CCT.

In our research, imaging at an energy level of 65 keV was the most accurate in differentiating malignant and benign nodules in both phases of contrast enhancement, with a sensitivity and specificity of 70% and 95%, respectively, for the adopted threshold value of 53.99 HU in AP, and 74% and 90%, respectively, for the threshold value of 49.65 HU in VP. The current literature considers VMI based on photon energy of 65–70 keV equivalent to standard CCT acquisition at 120 kVp [22]. The adopted threshold values of radiation attenuation at an energy level of 65 keV did not allow us to detect 14 malignant tumors in AP and 12 malignant tumors in VP for average NPVs (negative predictive values) of 0.56 and 0.58, respectively.

The degree of absorption of X-ray beams by the assessed tissue depends on its density, the type of radiation, and the amount of contrast medium accumulated in the tumor at the time of scanning. We obtained false negative results in nodules that absorbed smaller doses of radiation than expected, which means that these nodules accumulated less iodine in vessels and cellular spaces and indirectly indicates a lower degree of angiogenesis. The degree of angiogenesis indicates the degree of viability, degree of malignancy, and the vascularization sources [23,24]. Iodine, as the main component of a contrast medium, allows the assessment of vascular beds and intercellular spaces, and it facilitates the differentiation of lesions at various locations in the body based on the assumption that the malignant lesions exhibit a higher degree of contrast enhancement [25,26,27].

DECT allows the quantitative assessment of the concentration of iodine accumulated in a unit of tissue volume. Recent studies have showed the correlation of the iodine accumulation in the pulmonary nodule with the type of primary or metastatic tumor [28], pathological grades of non-small cell lung cancer [29,30], and tumor gene expression [31,32,33]. Son YJ et al. [30], analyzing the DECT results in patients with pure ground-glass opacity nodules or part solid nodules with solid components less than 5 mm in diameter, concluded that DECT with the quantitative analysis of iodine concentration parameters improved the power of diagnostic accuracy in distinguishing invasive adenocarcinoma from adenocarcinoma in situ or a minimally invasive one in comparison to results obtained in virtual non-contrast images (VNC) alone.

Some authors indicated the possible role of DECT iodine quantification in the evaluation of metabolic activity of the lung tumor. DECT iodine concentration was described as a possible substitute to 18F-FDG PET/CT in the evaluation of the lung tumor response to treatment [34,35], as an imaging biomarker of residual tumor vitality to predict tumor progression [36] or to predict the effects of chemotherapy in patients with advanced lung adenocarcinoma [37]. Ren et al. [34] indicated the possible role of iodine-related quantification in DECT in the evaluation of the lung cancer response to treatment (radiotherapy alone or chemoradiotherapy) as a feasible substitute to ^18^FDG PET-CT examination in such cases. The both methods reveled the decrees of the examined parameter: the iodine amount and FDG (fluorodeoxyglucose) uptake with tumor shrinking. Schmid-Bindert et al. [35], analyzing the results of DECT in the arterial phase and ^18^FDG PET-CT examinations in patients with lung cancer, found a strong correlation between the standardized uptake value (SUV_max_) of ^18^FDG PET-CT and the maximum iodine-related attenuation (IRA) in all lung tumors and in patients with non-small cell lung cancer (NSCLC), and a moderate correlation when the interval between both the studies was less than 21 days. In a group of 93 patients, Choe et al., by analyzing the radiomics features extracted from iodine overlay maps, concluded that spectral DECT can add prognostic information about overall survival and disease-free survival in patients with resectable lung cancer [38]. 

Our research demonstrated that the concentration of iodine in malignant tumors was significantly higher than that in benign lesions in AP and VP, which may have practical use in differential diagnosis. Zhang Y et al. obtained similar results in a comparable cohort [23,27]. The calculated iodine concentrations of 14.84 × 100 µg/cm^3^ in AP and 12.11 × 100 µg/cm^3^ in VP correctly diagnosed malignant tumors in 40/46 (in AP) and 44/46 (in VP) subjects (86% and 96%, respectively), with a sensitivity and specificity of 87% and 74%, respectively (AP), and 96% and 63%, respectively (VP). The most common pathology in patients with false-positive results (5 and 8 people with incorrectly diagnosed malignancy after the examination in AP and VP, respectively) was inflammatory infiltrates, which were diagnosed in all five patients examined in AP and 5 of the 7 patients misdiagnosed based on VP. Active inflammatory lesions with increased angiogenesis may mask the presence of a malignant tumor, which is a diagnostic difficulty with scant clinical data and lack of a prospective assessment. Hou et al. revealed that the normalized iodine concentration (NIC) in the central region of inflammatory mases in lung was higher than that in lung cancer, with the highest sensitivity (86%) and specificity (100%) in the venous phase. In our material, the low number of examined patients with inflammatory masses has not allowed us to make a proper analysis [39].

In our opinion, regardless of the higher radiation burden compared to conventional CT examination, DECT can enable the earlier diagnosis of slowly growing nodules. Higher iodine concentration in a tumor (as a result of increased angiogenesis) could manifest itself earlier than changes in glucose uptake represented by SUV value in PET/CT examination, thus making DECT especially useful in assessing small nodules. Taking into consideration the results of the cited publications, DECT (compared to PET/CT) could be a valuable tool not only in the initial diagnosis but also in the assessment of tumor treatment response, with a favorable lower cost and a similar or higher radiation exposure depending on the used examine protocol.

### Limitations

Due to the complex pulmonary vascular anatomy, the assessment of nodule enhancement in the lungs is not as unambiguous as in organs with a single blood supply.

Another limitation of the present study was the small number of examined cases, especially patients with benign tumors. However, our results suggest that additional research on a larger population would be beneficial.

The size of tumors was different in both groups. Malignant lesions were larger, and the difference was statistically significant. Malignant lesions exhibited a negative correlation between the size of the nodule and iodine accumulation [40]. In particular, analyses of lesions smaller than or equal to 8 mm in diameter may be useful in the early detection of lung cancer when correlated with risk factors, such as smoking, as per current recommendations [41]. The differentiation between the benign and malignant nodules in our research was based solely on histopathological results without taking into account other aspects, like lesion size, or epidemiological factors, such as nicotine use.

Another limitation is related to the propagation of regions of interest, which were not automatically copied between the AP and VP images. The manual copying of ROIs could lead to inaccurate readings. However, the ROIs from AP images were meticulously duplicated to the same areas in VP images in all cases to minimize errors.

Inflammatory lesions are a common reason for CT imaging, and it is difficult to unambiguously assess malignancy, as demonstrated in the present article. The authors support further research and analysis of this issue [5,23,39]. The combination of clinical data, risk factor assessment, qualitative and quantitative analyses of DECT and retrospective assessment of the lung tumor may improve diagnostic results in ambiguous cases.

The higher radiation burden of DECT compared to CCT means that this method is not routinely used in clinical practice, however DECT may demonstrate a clinical benefit in oncologic patients.

## 5. Conclusions

Virtual monochromatic images from dual-energy CT may be used as a tool in the differentiation of benign and malignant solitary pulmonary nodules smaller than 30 mm in diameter. Iodine concentrations in lung tumors allow the assessment of the malignancy of lung nodules, and the analysis of venous phase images results in the highest clinical usefulness. A single-phase DECT examination with scans acquired 90 s after contrast media injection is recommended.

## Figures and Tables

**Table 1:** Inclusion and exclusion criteria.

Patients	Exclusion Criteria
98 patients with a high risk of lung cancer, hospitalized between January 2017 and June 2018.	Exclusion criteria for CT examination:
	prior history of lung cancer (*n* = 5);
	contrast media hypersensitivity (*n* = 3);
	pregnancy (*n* = 0);
	kidney failure (*n* = 5);
	lack of patient’s consent (*n* = 1).
84 patients underwent DECT examinations to prospectively assess SPNs.	Exclusion criteria for analysis: lesion of a long-axis diameter larger than 30 mm (*n* = 8); “Ground-glass” lesion (*n* = 6); lack of histopathological confirmation of diagnosis (*n* = 4); coexistence of another cancer (*n* = 1).
65 patients whom we included in the statistical analysis were later split into two groups based on histopathological results: Patients with benign lesions; Patients with malignant lesions.	

**Table 2:** Nodules diagnoses and numbers.

Malignant	46 (71%)
Adenocarcinoma	23 (35%)
Squamous cell carcinoma	18 (28%)
Large cell neuroendocrine carcinoma	3 (4%)
Squamous cell (95%) and neuroendocrine carcinoma	1 (2%)
Small cell carcinoma	1 (2%)
Benign	19 (29%)
Inflammatory infiltrations	9 (14%)
Sarcoidosis	5 (8%)
Fibroma	2 (4%)
Hamartoma	1 (1%)
Hematoma	1 (1%)
Tuberculoma	1 (1%)

**Table 3:** Student’s *t*-test. Analyses of differences in IC maps between the malignant and benign nodules. Thresholds, sensitivity, specificity, and AUCs are based on the ROC curves in AP and VP.

Parameter	Malignant (*n* = 45)		Benign (*n* = 21)		T	*p* Value	Thresholds	Sensitivity (%)	Specificity (%)	AUC
	Mean	SD	Mean	SD						
**IC (AP)**	19.72	5.18	12.63	3.90	5.35	<0.001	14.84	87	74	0.859
**IC (VP)**	18.11	4.60	12.85	3.87	4.37	<0.001	12.11	96	63	0.817

**Table 4:** Diagnoses of lung tumors on 65 keV VMI maps and IC (iodine concentration) maps in the arterial (AP) and venous (VP) contrast enhancement phase, respectively, based on the Youden index of ROC analysis.

	Results	True Positives	False Positives	False Negatives	True Negatives	PPV	NPV	Youden
Parameters								
65-keV VMI *(AP)*		32	1	14	18	0.97	0.56	0.643
65-keV VMI *(VP)*		34	2	12	17	0.94	0.58	0.634
IC (AP)		40	5	6	14	0.89	0.70	0.606
IC (VP)		44	7	2	12	0.86	0.86	0.598

## Abbreviations and Acronyms

_SS_DECT	single source Dual Energy Computed Tomography
DECT	Dual Energy Computed Tomography
CCT	Conventional Computed Tomography
VMI	Virtual monochromatic image
VMS	Virtual Monochromatic Spectral
IC	Iodine concentration
SPN	Solitary pulmonary nodule
ROI	Region of interest
AP	Arterial phase enhancement
VP	Venous phase enhancement

## Appendix A

**Table A1:** Analysis of differences between malignant and benign nodules. Student’s *t* and Mann–Whitney U * analyses. Thresholds, sensitivity, specificity, and AUCs are based on the ROC curves for each keV energy level in AP.

Parameter keV (AP)	Malignant (*n* = 46)		Benign (*n* = 19)		*p*-Value	Thresholds	Sensitivity (%)	Specificity (%)	AUC
	Mean * Median	SD * Range	Mean * Median	SD * Range					
40	* 154.76	* 77.17–399.58	* 104.56	* 70.07–178.5	0.001	134.10	72	74	0.761
45	* 129.46	* 63.48–317.39	* 85.80	* 59.65–142.52	<0.001	106.08	74	74	0.783
50	* 109.70	* 52.36–251.17	* 68.42	* 51.26–113.57	<0.001	84.19	76	74	0.800
55	* 94.93	* 43.86–200.01	* 59.34	* 44.54–92.55	<0.001	93.99	54	100	0.827
60	* 80.87	* 35.33–151.58	* 47.61	* 35.25–75.57	<0.001	52.32	84	79	0.849
65	* 65.92	* 28.62–116.34	* 35.60	* 26.69–65.39	<0.001	53.99	70	95	0.852
70	* 59.16	* 25.78–95.00	* 32.67	* 21.19–57.62	<0.001	48.32	67	94	0.855
75	50.27	14.46	30.96	10.14	<0.001	43.38	67	94	0.851
80	45.83	12.87	28.03	10.30	<0.001	40.02	70	89	0.849
85	42.10	12.16	25.08	10.45	<0.001	38.51	67	95	0.854
90	38.13	11.47	22.04	10.58	<0.001	36.66	65	95	0.846
95	34.64	11.14	19.61	10.84	<0.001	32.32	65	89	0.824
100	32.23	11.00	17.66	11.10	<0.001	28.70	67	84	0.823
105	30.08	10.98	16.05	11.34	<0.001	27.83	60	89	0.811
110	28.06	11.04	14.54	11.59	<0.001	26.76	59	89	0.800
115	26.56	11.13	13.42	11.78	<0.001	23.31	63	84	0.791
120	25.14	11.25	12.37	11.97	<0.001	26.29	52	94	0.776
125	23.91	11.38	11.79	11.82	<0.001	26.25	50	95	0.768
130	22.89	11.52	10.69	12.30	<0.001	25.14	50	95	0.761
135	21.91	11.65	9.96	12.45	<0.001	23.39	52	95	0.757
140	21,3	11,98	9,45	12,47	<0.001	22.95	52	95	0.752

* non-normal distribution.

**Table A2:** Analysis of differences between malignant and benign nodules. Student’s *t* and Mann–Whitney U * analyses. Thresholds, sensitivity, specificity and AUCs are based on the ROC curves for each keV energy level in VP.

Parameter keV (VP)	Malignant (*n* = 46)		Benign (*n* = 19)		*p*-Value	Thresholds	Sensitivity (%)	Specificity (%)	AUC
	Mean * Median	SD * Range	Mean * Median	SD * Range					
40	* 147.41	* 84.96–249.13	* 112.69	* 69.41–196.97	0.004	121.89	76	69	0.728
45	* 124.67	* 73.38–200.04	* 92.09	* 60.62–154.43	0.002	98.32	76	69	0.743
50	* 105.46	* 64.09–157.44	* 74.13	* 53.52–121.20	<0.001	74.50	87	58	0.770
55	* 88.92	* 54.87–142.44	* 60.30	* 44.56–97.28	<0.001	61.86	91	58	0.800
60	73.38	18.88	51.34	10.79	<0.001	69.68	59	100	0.839
65	61.62	16.37	40.88	8.85	<0.001	49.65	74	90	0.858
70	54.90	14.57	36.03	8.38	<0.001	48.64	65	95	0.857
75	49.49	13.37	32.14	8.28	<0.001	40.27	74	89	0.855
80	45.49	12.62	29.45	8.14	<0.001	39.42	70	95	0.855
85	41.91	12.26	26.73	8.10	<0.001	37.29	70	95	0.850
90	38.45	12.17	23.86	8.15	<0.001	32.48	74	89	0.848
95	35.43	12.12	21.36	8.51	<0.001	32.59	63	95	0.833
100	32.99	12.14	19.35	8.86	<0.001	32.14	56	100	0.823
105	31.02	12.22	17.68	9.19	<0.001	30.06	56	100	0.822
110	29.16	12.32	16.12	9.53	<0.001	28.55	59	100	0.814
115	* 28.87	* 1.80–71.22	* 15.92	* −4.31–27.66	<0.001	27.77	59	100	0.807
120	* 27.23	* −0.24–70.49	* 15.10	* −6.37–27.28	<0.001	26.72	56	95	0.793
125	* 26.47	* −1.92–69.87	* 14.36	* −8.14–26.88	<0.001	25.77	54	95	0.793
130	* 25.88	* −3.29–69.35	* 13.76	* −9.60–26.59	<0.001	26.98	48	100	0.789
135	* 25.34	* −4.71–68.88	* 13.22	* −11.03–26.25	<0.001	22.06	63	84	0.785
140	* 24.78	* −5.82–68.49	* 12.74	* −12.30–26.03	<0.001	24.70	53	95	0.780

* non-normal distribution.
